# A telomere‐to‐telomere haplotype‐resolved genome of white‐fruited strawberry reveals the complexity of fruit colour formation of cultivated strawberry

**DOI:** 10.1111/pbi.14479

**Published:** 2024-09-20

**Authors:** Junxiang Zhang, Shuang Liu, Shuo Zhao, Yuxin Nie, Zhihong Zhang

**Affiliations:** ^1^ Liaoning Key Laboratory of Strawberry Breeding and Cultivation College of Horticulture, Shenyang Agricultural University Shenyang China

**Keywords:** cultivated strawberry, telomere‐to‐telomere, fruit colour, *FaMYB10*, homoeolog expression

Cultivated strawberry (*Fragaria* × *ananassa*, 2*n* = 8*x* = 56) is an important horticultural crop with substantial economic and nutritional value. The improvement of cultivated strawberry is more challenging not only in its octoploid genome but also in the frequent homoeologous exchanges and polyploidization, which replaces substantial portions of some subgenomes with sequences derived from ancestrally related chromosomes (Edger *et al*., 2019). Therefore, a high‐quality genome for the cultivated strawberry will provide important information for identifying agriculturally important genes for breeding. Several cultivated strawberry genomes have been assembled. However, some published reference genomes of cultivated strawberries remained incomplete, and some published genomes of cultivated strawberries were not truly haplotype‐resolved (Edger *et al*., 2019; Lee *et al*., 2021; Mao *et al*., 2023; Song *et al*., 2024).

Here, we de novo assembled a telomere‐to‐telomere haplotype‐resolved reference genome with 56 chromosomes (Figure 1a) of the white‐fruited strawberry cultivar ‘Chulian’ (Figure S1) by incorporating PacBio HiFi, ONT ultra‐long and Hi‐C sequencing, and Illumina sequencing data. The centromere candidate sequences and regions of each chromosome were identified (Figure S2 and Table S1). We divided 56 chromosomes into two haplotypes, Hap1 (chr × − × −1) and Hap2 (chr × − × −2), and each haplotype includes 28 chromosomes. The final genome assembly sizes were 787.52 Mb with 33 contigs for Hap1 and 778.03 Mb with 34 contigs for Hap2, respectively. The contigs N50 of Hap1 and Hap2 were 27.92 Mb and 26.45 Mb, respectively. We identified 52 telomeres in Hap1 and 50 in Hap2 by investigating telomeric repeats (TTTAGGG)n (Figures S2; Table S2).

**Figure 1:**
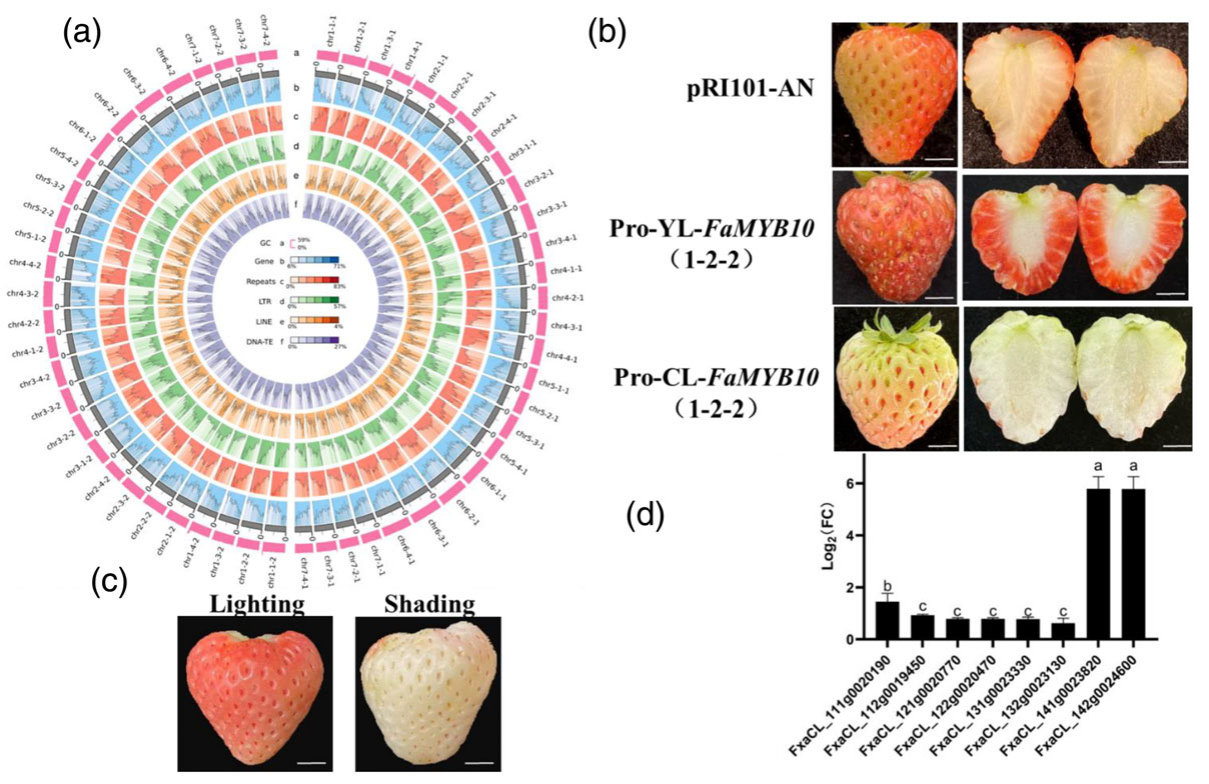
Genomic features and the loss‐of‐anthocyanin phenotype of ‘Chulian’ strawberry. (a) The haplotype‐resolved genome assembly of ‘Chulian’ strawberry. (b) Transient functional analysis of point mutation of ‘Chulian’ strawberry *FaMYB10* on chr1‐2‐2. Scale, 1 cm. (c) The phenotype of ‘Chulian’ strawberry under lighting and shading treatment. Scale, 1 cm. (d) The fold change of expression levels of *FaMYB10* in ‘Chulian’ strawberry under lighting and shading treatment. [Correction added on 23 September 2024, after first online publication: new Figure 1 and caption is updated in this version.]

The integrity and accuracy of the genome assembly of ‘Chulian’ were evaluated by Benchmarking Universal Single‐Copy Orthologs (BUSCO) assessments (Tables S3 and S4) and showed that the genome assembly of ‘Chulian’ had high coverage and quality. A total of 110 001 and 108 859 protein‐coding genes were annotated in the Hap1 and Hap2, respectively. In addition, 5864 and 5830 transcription factors were predicted in the Hap1 and Hap2, respectively. The information on repetitive sequences is in Tables S5 and S6.

We conducted collinearity analysis of Hap1 (Reference) and Hap2 (Query) to investigate variations of two haplotype genomes of ‘Chulian’ strawberry. We discovered 16 315 syntenic blocks totaling ~631 Mb, covering 92.82% and 93.96% of the Hap1 and Hap2 genomes (Figure S3; Table S7). Moreover, we compared ‘Chulian’ with the high‐quality cultivated strawberry ‘Yanli’ (Mao *et al*., 2023) due to their diverse phenotype differences, such as fruit colour, hardness and powdery mildew resistance. The comparison results showed that the haplotype genome of ‘Chulian’ and ‘Yanli’ had high similarity and collinearity (Figure S4). We compared Hap1 and Hap2 of ‘Chulian’ to Hap1 and Hap2 of ‘Yanli’ to analyse the number of structural variations (SVs), the length range of SVs and the position of the maximum SVs per chromosome (Figure S5). The SVs with lengths over 100 bp and located in the genomic gene regions (exons and introns), promoter region (2 kb from start codon) and downstream regions (2 kb from stop codon) between ‘Chulian’ and ‘Yanli’ had also been completely identified (Appendix S1). Interestingly, many genes of ‘Chulian’ with large SVs in their exon and promoter regions were related to disease resistance, including receptor protein kinase containing LRR repeats, TIR‐NBS‐LRR class protein, chitinase and putative powdery mildew resistance protein compared with ‘Yanli’ (Appendix S2). Moreover, we also found numerous transcription factors (WRKY, MYB, MADS‐box, bHLH, ERF, bZIP, etc.) of ‘Chulian’ with large SVs in these exon and promoter regions compared with ‘Yanli’ (Appendix S2), and the functions of these transcription factors need to be investigated in further.

The fruit flesh of ‘Chulian’ was white due to the loss of anthocyanin accumulation. To identify candidate genes responsible for the white fruit phenotype of ‘Chulian’, we examined the master positive regulator *FaMYB10* of anthocyanin biosynthesis in ‘Chulian’ and ‘Yanli’ by utilizing the high‐quality genomic sequence. Interestingly, the *FaMYB10* on chr1‐2‐1 had 8‐bp ‘ACTTATAC’ insertion in the 491 nucleotides of ‘Chulian’ (Figure S6a). The *FaMYB10* on chr1‐2‐1 of ‘Chulian’ germinated a truncated protein with 179 amino acids due to a premature stop codon relative to ‘Yanli’ (producing 233 amino acids; Figure S6b). The *FaMYB10* on chr1‐2‐2 only had a single nucleotide difference compared with ‘Yanli’. The point mutation (C to A) was found at the 94th nucleotide, resulting in an amino acid substitution from histidine (H) in ‘Yanli’ to asparagine (N) in ‘Chulian’ (Figures S1a, b). The transient functional analysis found that overexpression of *FaMYB10* on chr1‐2‐1 of ‘Yanli’ could restore the anthocyanin deficiency phenotype of ‘Chulian’ (Figure S7). Interestingly, the transient functional analysis found that the fruits of importing *FaMYB10* on chr1‐2‐2 of ‘Chulian’ with its promoter [Pro‐CL‐FaMYB10(1–2‐2)] did not restore the anthocyanin deficiency phenotype of ‘Chulian’. In contrast, the fruits of importing *FaMYB10* on chr1‐2‐2 of ‘Yanli’ with its promoter [Pro‐YL‐FaMYB10(1–2‐2)] recovered the anthocyanin deficiency phenotype of ‘Chulian’ (Figure 1b). Furthermore, some anthocyanin biosynthetic genes' expression levels increased in the fruits of importing Pro‐YL‐FaMYB10(1–2‐2) compared with the control fruit (Figure S8). These results suggested that the point mutation of *FaMYB10* on chr1‐2‐2 of ‘Chulian’ affected its function, and the molecular basis awaits further investigation. Together, 8‐bp insertion in *FaMYB10* on chr1‐2‐1 and the point mutation in *FaMYB10* on chr1‐2‐2 were the main reasons for the white fruit phenotype of the ‘Chulian’ strawberry.

Cultivated strawberry is an allo‐octoploid species with four subgenomes (Edger *et al*., 2019). Genes from different subgenomes display expression differences, and the dominant gene expression pattern is detected in many allopolyploid species. During the development of ‘Chulian’ strawberry fruits, *FaMYB10* on chr1‐2 was the dominant expression gene (Figure S9). However, the fruit skin of ‘Chulian’ turned red and accumulated anthocyanin under light treatment (Figure 1c). We conducted RNA‐seq of fruit skin of ripening fruits under lighting and shading treatments. A total of 5265 genes were differentially expressed. 2215 were upregulated, and 3050 were downregulated (Figure S10a). KEGG analysis revealed these differentially expressed genes mainly involved in plant hormone signal transduction, plant circadian rhythm, protein processing in the endoplasmic reticulum and flavonoid metabolism pathways (Figure S10b). Intriguingly, we found the transcript level of *FaMYB10* on chr1‐4 of ‘Chulian’ other than *FaMYB10* on chr1‐2 in the fruit skin under lighting treatment was significantly increased compared with fruit skin under shading treatment (Figure 1d). Moreover, we found the promoter of *FaMYB10* on chr1‐4 included more light‐responsive elements and salicylic acid and methyl jasmonate elements (Figure S11; Table S8) than *FaMYB10* on chr1‐2 of ‘Chulian’.

In conclusion, we obtained a high‐quality haplotype‐resolved genome of the octoploid white‐fruited cultivar ‘Chulian’. We found that an 8‐bp insertion in the coding region of *FaMYB10* on chr1‐2‐1 and the single nucleotide mutation in *FaMYB10* on chr1‐2‐2 were related to the loss of anthocyanins in the fruits. Interestingly, we found that the accumulation of anthocyanins was light‐regulated by activating the expression of *FaMYB10* on chr1‐4 instead of the dominant homoeologous *FaMYB10* on chr1‐2 during fruit development. These results will lay a solid foundation for comparative genomic analysis, understanding the expression pattern of genes in the subgenome of polyploidy species and fruit colour breeding of cultivated strawberry.

## Conflict of interest

The authors declare that they have no competing interests.

## Author contributions

Zhihong Zhang designed the experiments. Shuang Liu collected plant materials. Junxiang Zhang, Shuang Liu, Shuo Zhao and Yuxin Nie conducted experiments and analysed data. Junxiang Zhang and Zhihong Zhang wrote and modified the manuscript. All authors in this study read and approved the manuscript.

## Supporting information


**Appendix S1** The SVs with lengths over 100 bp and located in the genomic gene regions between ‘Chulian’ and ‘Yanli’.

**Appendix S2** The SVs with lengths over 100 bp and located in the upstream and exon of genes between ‘Chulian’ and ‘Yanli’.

**Appendix S3** Supplemental materials and methods.

**Figure S1** The phenotypes of ‘Chulian’ strawberry and the comparisons of the coding region and amino acids of FaMYB10 on chr1‐2‐2 from ‘Chulian’ and ‘Yanli’ strawberry.
**Figure S2** The contig numbers, telomeres, and centromeres of ‘Chulian’ strawberry genome.
**Figure S3** The collinearity of Hap1 and Hap2 of ‘Chulian’ strawberry genome.
**Figure S4** The collinearity of ‘Chulian’ and ‘Yanli’ strawberry genome.
**Figure S5** The structural variations (SVs) between the ‘Chulian’ and ‘Yanli’ strawberry genome.
**Figure S6** The comparisons of the coding region and amino acids of FaMYB10 on chr1‐2‐1 from ‘Chulian’ and ‘Yanli’ strawberry.
**Figure S7** The transient functional analysis of overexpression of *FaMYB10* on chr1‐2‐1 of ‘Yanli’ with CaMV 35S promoter on the fruits of ‘Chulian’.
**Figure S8** The expression of anthocyanin biosynthetic genes between the fruits of importing *FaMYB10* on chr1‐2‐2 of ‘Yanli’ with its promoter [Pro‐YL‐FaMYB10(1‐2‐2)] and importing *FaMYB10* on chr1‐2‐2 of ‘Chulian’ with its promoter [Pro‐CL‐FaMYB10(1‐2‐2)].
**Figure S9** The expression of *FaMYB10* from ‘Chulian’ strawberry fruit at green, white, and turn stage.
**Figure S10** The volcano map and KEGG of differentially expressed genes of ‘Chulian’ strawberry fruit under lighting and shading.
**Figure S11** The schematic figure of cis‐elements differences in the promoter region between *FaMYB10* on chr1‐2 and *FaMYB10* on chr1‐4 of ‘Chulian’ strawberry under lighting and shading treatment.
**Table S1** The Centromere information of the ‘Chulian’ strawberry genome.
**Table S2** The telomere information of the ‘Chulian’ strawberry genome.
**Table S3** Genome assembly integrity assessment of Hap1 of ‘Chulian’ strawberry by BUSCO.
**Table S4** Genome assembly integrity assessment of Hap2 of ‘Chulian’ strawberry by BUSCO.
**Table S5** The information on repetitive sequences of Hap1 of ‘Chulian’ strawberry.
**Table S6** The information on repetitive sequences of Hap2 of ‘Chulian’ strawberry.
**Table S7** The structural variations between Hap1 and Hap2 of ‘Chulian’ strawberry Genome.
**Table S8** The cis‐elements differences in the promoter region between *FaMYB10* on chr1‐2 and *FaMYB10* on chr1‐4 of ‘Chulian’ strawberry under lighting and shading treatment.

## Data Availability

The genome data have been deposited in the Genome Warehouse in China National Genomics Data Center under the BioProject accession number PRJCA027111 and the Biosample number SAMC3827938.
