# Supplementary material for: A telomere‐to‐telomere haplotype‐resolved genome of white‐fruited strawberry reveals the complexity of fruit colour formation of cultivated strawberry
Source: Plant Biotechnol J. 2024 Sep 20;23(1):78–80. doi: 10.1111/pbi.14479 (PMC11672740; doi:10.1111/pbi.14479)
Supplement: Supplementary file 3 — Appendix S3 Supplemental materials and methods. [file PBI-23-78-s004.docx]

**Appendix S3.**

**Materials and Methods**

**Plant materials**

The octoploid cultivated strawberry (*Fragaria* × *ananassa*) cultivar ‘Chulian’ (2*n* = 8*x* = 56) was applied for genome sequencing. Another cultivated strawberry cultivar ‘Yanli’ was used for comparative genomic analysis and fruit color gene identification, and the genome data of ‘Yanli’ was uploaded in GDR (https://www.rosaceae.org/Analysis/14723107). The fruit of ‘Chulian’ is white, and the fruit of ‘Yanli’ is red. In addition, the fruit skin of ‘Chulian’ can turn light red under lighting conditions. The different developmental stages of ‘Chulian’ fruits at the big green, white, and turning stages were used for transcriptome analysis.

**MGI short-read sequencing and genome size and heterozygosity analysis**

The genomic DNA of ‘Chulian’ was obtained from young leaf tissue. The library was constructed by MGIEasy Universal DNA Library Prep Kit V1.0（MGI Tech Co., Ltd., Shenzhen, China). The raw sequencing data was acquired through the DNBSEQ-T7RS platform (MGI Tech Co., Ltd., Shenzhen, China) and then processed with FastQC version 0.11.3 (Andrews et al., 2015) to remove adapters and low-quality bases. Clean data were applied for the genome assembly assessment and error correction of ‘Chulian’ genome assembly. The genome size and heterozygosity of ‘Chulian’ were estimated based on k-mer analysis. The k-mer (k = 75) depth distribution curve was produced by GCE version 1.0.0 (Liu et al., 2013). The ‘Chulian’ genome size was estimated utilizing the following formula: GS = k-mer number/average k-mer depth (Gao et al. 2018). The heterozygosity was calculated based on the formula described by Liu et al. (2013).

**PacBio SMRT and ONT ultra-long read sequencing and assembly**

High-quality genomic DNA of ‘Chulian’ was used for the library construction. The SMRT Bell library was carried out based on the manufacturer's protocol (Pacific Biosciences, CA, USA) and (Oxford Nanopore Technologies, Oxford, UK). The library was sequenced via PacBio Sequel II and PromethION sequencer, respectively. The Hifiasm pipeline (https://github.com/chhylp123/hifiasm) was used for the *de novo* assembly ‘Chulian’.

**Hi-C assembly**

To anchor sequences to the chromosomes, we prepared the Hi-C library by the Illumina HiSeq platform. HI-C data was mapped and processed by the HiCUP pipeline (Wingett et al., 2015). 3D *de novo* assembly (3D-DNA) pipeline was used for germinating chromosome-length scaffolds (Dudchenko et al., 2017). The juicer pipeline was used to construct a Hi-C interaction map, and JuiceBox pipeline was applied for the corrections of orders, orientations, and mis-joins (Durand et al., 2016).

**Telomere and centromere predictions**

The telomeres of ‘Chulian’ strawberry genome assembly were identified by investigating telomeric repeats (TTTAGGG)n. We utilized the quarTeT toolkit (Lin et al., 2023) to predict the centromere regions of the ‘Chulian’ strawberry genomes. A tandem repeat sequence with a length 147bp was identified in the centromere candidate regions.

**Evaluation of assembly quality**

The short reads from the second-generation sequencing and long reads from third-generation sequencing were aligned with the assembled genome of ‘Chulian’ by BWA version 0.7.12-r1039 (Li and Durbin 2009) and minimap2 version 2.22_x64-linux (Li, 2018) pipelines to perform sequence similarity evaluation, respectively. The BUSCO version 5.3.1 was used to analyze the completeness of the genome assembly (Manni et al., 2021). The Samtools version 1.9 (Li et al., 2009) and Picard version 1.124 (https://broadinstitute.github.io/picard/) pipelines were applied for the accuracy and heterozygosity analysis. The homozygous/heterozygous SNP and InDel rates were tested via GATK version 4.2.0.0 (McKenna et al., 2010).

**Identification of** **repetitive sequences and transcription factors**

Repetitive sequences were identified through the integration of *de novo* predictions and homology-based predictions. Tandem repeats finder (TRF) version 4.09 (Benson et al., 1999) with default parameters was used to *de novo* predictions of repetitive sequences. Homology-based predictions of repetitive sequences were acquired based on the Repbase database (Bao et al. 2015) using RepeatMasker version open-4.0.9 with default parameters (https://www.repeatmasker.org/). In addition, a *de novo* repetitive sequences database was constructed based on a combination of RepeatModeler version open-1.0.11 (Price et al. 2005) and LTR_FINDER_parallel version 1.0.7 (Ou et al. 2019), then RepeatMasker was applied to de novo predictions of repetitive sequences with default parameters. The final repetitive sequences were obtained by removing the overlapping parts of *de novo* predictions and homology-based predictions. Transcription factors were identified based on the plant transcription factor database (PlantTFDB v5.0; Tian et al., 2019).

**Gene prediction and functional annotations**

An integration of homology-based prediction, *de novo* prediction, and RNA-seq data was applied for annotations of protein-coding genes in the ‘Chulian’ genome. GlimmerHMM version 3.0.4 (Majoros et al. 2004) and Augustus version 3.3.2 (Stanke et al., 2006) were used for the *de novo* gene prediction. Sequences of *F. vesca* and *F.* × *ananassa* cultivar ‘Camarosa’, and ‘Yanli’ were used for identifying homologous genes by Liftoff version 1.6.3 under default parameters (Shumate et al. 2021). Trans ORF pipeline was used for gene prediction based on the RNA-seq data. All gene predictions via the above methods were integrated using Maker2 vertion 2.31.10 (Holt and Mark 2011) with default parameters to produce a final gene prediction.

Functional annotations were conducted through diamond version 2.0.14 pipeline with the parameters (--evalue 1e-05; Buchfink et al., 2021) to align with multiple databases, including the NR, SwissProt, TrEMBL, KEGG, and KOG databases (Kanehisa et al. 2016). InterProScan version 5.61-93.0 with the parameters (--seqtype p --formats TSV --goterms --pathways -dp ; Jones et al. 2014) was used to get motif information. Finally, predicted genes were performed KEGG, KOG, and GO functional and pathway enrichment analysis.

**Prediction of non-coding RNA and pseudogenes**

The tRNAscan-SE program (version 1.3.1) with the default parameters was used to identify tRNAs in the assembled ‘Chulian’ genome (Lowe and Eddy 1997). Due to the high conservatism of rRNAs among different species, rRNAs from closed-related species were selected as reference sequences and then blasted with ‘Chulian’ genome to identify the rRNAs of the ‘Chulian’ genome. INFERNAL software (Nawrocki and Eddy, 2013) was applied to identify miRNAs and snRNAs against the Rfam 14.10 database (Kalvari et al., 2021)

**Transient transformation of strawberry fruit**

Transient transformation of strawberry fruit was based on pRI101-AN vector. For transient complementation test vector construction, the coding region of ‘Yanli’ strawberry *FaMYB10* on chr1-2-1 with 35S promoter was introduced into pRI101-AN vector [named as 35S-FaMYB10 (1-2-1)]. For transient functional analysis of point mutation of ‘Chulian’ strawberry, *FaMYB10* on chr1-2-2 of ‘Chulian’ with its promoter [named as Pro-CL-FaMYB10(1-2-2)] and *FaMYB10* on chr1-2-2 of ‘Yanli’ with its promoter [named as Pro-YL-FaMYB10(1-2-2)] were introduced into pRI101-AN vector. A single colony of the bacterial strain containing 35S-FaMYB10 (1-2-1), Pro-CL-FaMYB10(1-2-2), and Pro-YL-FaMYB10(1-2-2) were transferred into liquid yeast extract peptone (YEP) medium supplemented with 50 mg/L rifampicin and 50 mg L^-1^ kanamycin, respectively. Then, the bacterial solution was added to 50 mL YEP and cultivated at 28℃ with shaking for 6 to 8 h until the OD600 was between 0.8 to 1.0. The *Agrobacterium* culture was centrifuged at 5000 rpm for 6 min, and the pellet was resuspended in MS (Murashige and Skoog 1962) medium containing MS basal medium, 30g/L sucrose, and 1mol/L KOH in a 50 mL centrifuge tube. The final OD600 of this mixture was about 0.8. The fruit of ‘Chulian’ at about 12 days (big green stage) after pollination was used for transient transformation.

**Quantitative RT-PCR analysis**

Total RNA from fruit was obtained utilizing the modified CTAB method as described by Wang et al. (2023). The cDNA synthesis, the steps for Quantitative RT-PCR (RT-qPCR) analysis, and the method for relative mRNA levels analysis were the same as the method described by Mao et al. (2024). The 2^-ΔΔCt^ method was employed to analyze the relative mRNA levels. All data were normalized first with the level of the *FaGAPC2* internal transcript control (Mao et al., 2024) and then with the expression of controls. Each sample was tested in triplicate with three biological replicates.

**Statistical analyses**

Statistical analysis was conducted using SPSS 19.0 software (International Business Machines Corporation, New York, USA). The significance of differences was analyzed by a one-way ANOVA test (p<0.05).

**References**

Andrews Simon, A. FastQC (2015) A quality control tool for high throughput sequence data.

Bao W, Kojima KK, Kohany O (2015) Repbase Update, a database of repetitive elements in eukaryotic genomes. *Mobile DNA* **6** (1):11.

Benson Gary (1999) Tandem repeats finder: a program to analyze DNA sequences. *Nucleic Acids Research* **27** (2):573-580.

Buchfink B, Klaus Rr, Hajk-Georg D (2021) Sensitive protein alignments at tree-of-life scale using DIAMOND. *Nature Methods* **18** (4):366-368.

[Dudchenko O](https://ss.zhizhen.com/s?sw=author(Dudchenko,+Olga)), [Batra SS](https://ss.zhizhen.com/s?sw=author(Batra,+Sanjit+S)), [Omer AD](https://ss.zhizhen.com/s?sw=author(Omer,+Arina+D)), [Nyquist SK](https://ss.zhizhen.com/s?sw=author(Nyquist,+Sarah+K)), [Hoeger M](https://ss.zhizhen.com/s?sw=author(Hoeger,+Marie)), [Durand NC](https://ss.zhizhen.com/s?sw=author(Durand,+Neva+C)), *et al*. (2017) De novo assembly of the Aedes aegypti genome using Hi-C yields chromosome-length scaffolds. *Science* **356** (6333):92-95.

Gao F, Wang X, Li X, Xu M, Li H, Abla M, Sun H, *et al*. (2018) Long-read sequencing and de novo genome assembly of *Ammopiptanthus nanus*, a desert shrub. *GigaScience* **7** (7):giy074.

Holt Carson, Mark Yandell (2011) MAKER2: an annotation pipeline and genome-database management tool for second-generation genome projects. *BMC Bioinformatics* **12** (1):1-14.

Jones P, Binns D, Chang HY, Fraser M, Li W, McAnulla C, McWilliam H, *et al*. (2014). InterProScan 5: genome-scale protein function classification. *Bioinformatics*, **30**(9), 1236-1240.

[Kalvari I](https://ss.zhizhen.com/s?sw=author(Kalvari,+Ioanna%3csup%3e1%3c/sup%3e)), [Nawrocki EP,](https://ss.zhizhen.com/s?sw=author(Nawrocki,+Eric+P.%3csup%3e2%3c/sup%3e)) [Ontiveros-Palacios N](https://ss.zhizhen.com/s?sw=author(Ontiveros-Palacios,+Nancy%3csup%3e1%3c/sup%3e)), [Argasinska J](https://ss.zhizhen.com/s?sw=author(Argasinska,+Joanna%3csup%3e1%3c/sup%3e)), [Lamkiewicz K](https://ss.zhizhen.com/s?sw=author(Lamkiewicz,+Kevin%3csup%3e3%3c/sup%3e%3csup%3e,d%3c/sup%3e)), [Marz M, d](https://ss.zhizhen.com/s?sw=author(Marz,+Manja%3csup%3e3%3c/sup%3e%3csup%3e,d%3c/sup%3e))[Griffiths-JS](https://ss.zhizhen.com/s?sw=author(Griffiths-Jones,+Sam%3csup%3ee%3c/sup%3e)), *et al*. (2021) Rfam 14: expanded coverage of metagenomic, viral and microRNA families. *Nucleic Acids Research* **49**(D1): D192-D200.

Kanehisa M, Sato Y, Kawashima M, Furumichi M, & Tanabe M. (2016). KEGG as a reference resource for gene and protein annotation. *Nucleic Acids Research*, **44(**D1), D457-D462.

Li H. (2018). Minimap2: pairwise alignment for nucleotide sequences. *Bioinformatics (Oxford, England*) **34** (18):3094–3100.

Li H and Durbin R. (2009). Fast and accurate short read alignment with Burrows-Wheeler transform. *Bioinformatics*, **25**(14), 1754-1760.

[Li H](https://ss.zhizhen.com/s?sw=author(Li,+H+(Li,+Heng)%3csup%3e1%3c/sup%3e+)), [Handsaker B,](https://ss.zhizhen.com/s?sw=author(+Handsaker,+B+(Handsaker,+Bob)%3csup%3e2,3%3c/sup%3e+)) [Wysoker A](https://ss.zhizhen.com/s?sw=author(+Wysoker,+A+(Wysoker,+Alec)%3csup%3e2,3%3c/sup%3e+)), [Fennell T,](https://ss.zhizhen.com/s?sw=author(+Fennell,+T+(Fennell,+Tim)%3csup%3e2,3%3c/sup%3e+)) [Ruan J,](https://ss.zhizhen.com/s?sw=author(+Ruan,+J+(Ruan,+Jue)%3csup%3e4%3c/sup%3e+)) [Homer N,](https://ss.zhizhen.com/s?sw=author(+Homer,+N+(Homer,+Nils)%3csup%3e5%3c/sup%3e+)) [Marth G,](https://ss.zhizhen.com/s?sw=author(+Marth,+G+(Marth,+Gabor)%3csup%3e6%3c/sup%3e+)) *et al*. (2009) The sequence alignment/map format and SAMtools. *Bioinformatics* **25** (16): 2078-2079.

Lin Y, Ye C, Li X, Chen Q, Wu Y, Zhang F, Pan R, Zhang S, Chen S, Wang X, Cao S, Wang Y, Yue Y, Liu Y, Yue, J. (2023) quarTeT: a telomere-to-telomere toolkit for gap free genome assembly and centromeric repeat identification. *Horticulture research* **8**:122-128.

Liu BH, Shi YJ, Yuan JY, Hu XS, Zhang H, Li N (2013) Estimation of genomic characteristics by analyzing K-mer frequency in de novo genome projects. *Quantitative Biology* **35 :**62-67.

Lowe TM, Eddy SR (1997) tRNAscan-SE: a program for improved detection of transfer RNA genes in genomic sequence. *Nucleic Acids Research* **25** (5):955-964.

Majoros WH, Pertea M, Salzberg SL (2004) TigrScan and GlimmerHMM: two open source ab initio eukaryotic gene-finders. *Bioinformatics* **20** (16):2878-2879.

Manni M, Berkeley MR, Seppey M, Simão FA, Zdobnov EM (2021) BUSCO update: novel and streamlined workflows along with broader and deeper phylogenetic coverage for scoring of eukaryotic, prokaryotic, and viral genomes. *Molecular Biology and Evolution* **38** (10):4647–4654.

Mao J, Li J, Wang Y, Zhang Z. (2024) Selection and validation of reference genes for qRT-PCR in cultivated octoploid strawberry. *Fruit Research* **4** :e010

McKenna A, Hanna M, Banks E, Sivachenko A, Cibulskis K, Kernytsky A, Garimella K, *et al*. (2010) The Genome Analysis Toolkit: a MapReduce framework for analyzing next-generation DNA sequencing data. *Genome Research* **20** (9):1297–303.

Nawrocki EP, Eddy SR (2013) Infernal 1.1:100-fold faster RNA homology searches. *Bioinformatics* **29** (22):2933-2935.

Durand NC, Shamim MS, Machol I, Rao SS, Huntley MH, Lander ES, Aiden EL. (2016). Juicer provides a one-click system for analyzing loop-resolution Hi-C experiments. *Cell Systems* **3** (1):95-98.

Ou S, Ning J (2019) LTR_FINDER_parallel: parallelization of LTR_FINDER enabling rapid identification of long terminal repeat retrotransposons. *Mobile DNA* **10** (1):1-3.

Price AL, Jones NC, & Pevzner PA. (2005). De novo identification of repeat families in large genomes. *Bioinformatics*, **21**(Suppl 1), i351–i358.

Shumate Alaina, Steven L Salzberg (2021) Liftoff: accurate mapping of gene annotations. *Bioinformatics* **37 (**12):1639-1643.

[Stanke M](https://ss.zhizhen.com/s?sw=author(Stanke,+M)), [Keller O](https://ss.zhizhen.com/s?sw=author(Keller,+O)), [Gunduz I](https://ss.zhizhen.com/s?sw=author(Gunduz,+I)) (2006) AUGUSTUS: ab initio prediction of alternative transcripts. *Nucleic Acids Research* **34** (suppl_2):W435-W439.

Tian F, Yang DC, Meng YQ, Jin JP and Gao G (2019) PlantRegMap: charting functional regulatory maps in plants. *Nucleic Acids Research*, **48**(D1), D1104-D1113.

Wang B, Li W, Xu K, Lei Y, Zhao D, Li X, Zhang J, Zhang Z. (2023). A splice site mutation in the *FvePHP* gene is associated with leaf development and flowering time in woodland strawberry. *Horticulture Research*, **10**(1), uhac249.

[Wingett S](https://ss.zhizhen.com/s?sw=author(Wingett,+Steven)), [Ewels P](https://ss.zhizhen.com/s?sw=author(Ewels,+Philip)), [Furlan-Magaril M](https://ss.zhizhen.com/s?sw=author(Furlan-Magaril+Mayra)), [Nagano T](https://ss.zhizhen.com/s?sw=author(Nagano+Takashi)), [Schoenfelder S](https://ss.zhizhen.com/s?sw=author(Schoenfelder,+Stefan)), [Fraser P](https://ss.zhizhen.com/s?sw=author(Fraser,+Peter)), [Andrews S](https://ss.zhizhen.com/s?sw=author(Andrews,+Simon)) (2015) HiCUP: pipeline for mapping and processing Hi-C data. *F1000Research* **4**:1310.
