# Supplementary material for: A telomere‐to‐telomere haplotype‐resolved genome of white‐fruited strawberry reveals the complexity of fruit colour formation of cultivated strawberry
Source: Plant Biotechnol J. 2024 Sep 20;23(1):78–80. doi: 10.1111/pbi.14479 (PMC11672740; doi:10.1111/pbi.14479)
Supplement: Supplementary file 4 — Figure S1 The phenotypes of ‘Chulian’ strawberry and the comparisons of the coding region and amino acids of FaMYB10 on chr1‐2‐2 from ‘Chulian’ and ‘Yanli’ strawberry. Figure S2 The contig numbers, telomeres, and centromeres of ‘Chulian’ strawberry genome. Figure S3 The collinearity of Hap1 and Hap2 of ‘Chulian’ strawberry genome. Figure S4 The collinearity of ‘Chulian’ and ‘Yanli’ strawberry genome. Figure S5 The structural variations (SVs) between the ‘Chulian’ and ‘Yanli’ strawberry genome. Figure S6 The comparisons of the coding region and amino acids of FaMYB10 on chr1‐2‐1 from ‘Chulian’ and ‘Yanli’ strawberry. Figure S7 The transient functional analysis of overexpression of FaMYB10 on chr1‐2‐1 of ‘Yanli’ with CaMV 35S promoter on the fruits of ‘Chulian’. Figure S8 The expression of anthocyanin biosynthetic genes between the fruits of importing FaMYB10 on chr1‐2‐2 of ‘Yanli’ with its promoter [Pro‐YL‐FaMYB10(1‐2‐2)] and importing FaMYB10 on chr1‐2‐2 of ‘Chulian’ with its promoter [Pro‐CL‐FaMYB10(1‐2‐2)]. Figure S9 The expression of FaMYB10 from ‘Chulian’ strawberry fruit at green, white, and turn stage. Figure S10 The volcano map and KEGG of differentially expressed genes of ‘Chulian’ strawberry fruit under lighting and shading. Figure S11 The schematic figure of cis‐elements differences in the promoter region between FaMYB10 on chr1‐2 and FaMYB10 on chr1‐4 of ‘Chulian’ strawberry under lighting and shading treatment. Table S1 The Centromere information of the ‘Chulian’ strawberry genome. Table S2 The telomere information of the ‘Chulian’ strawberry genome. Table S3 Genome assembly integrity assessment of Hap1 of ‘Chulian’ strawberry by BUSCO. Table S4 Genome assembly integrity assessment of Hap2 of ‘Chulian’ strawberry by BUSCO. Table S5 The information on repetitive sequences of Hap1 of ‘Chulian’ strawberry. Table S6 The information on repetitive sequences of Hap2 of ‘Chulian’ strawberry. Table S7 The structural variations between Hap1 and Hap2 of ‘Chulian’ s [file PBI-23-78-s001.docx]

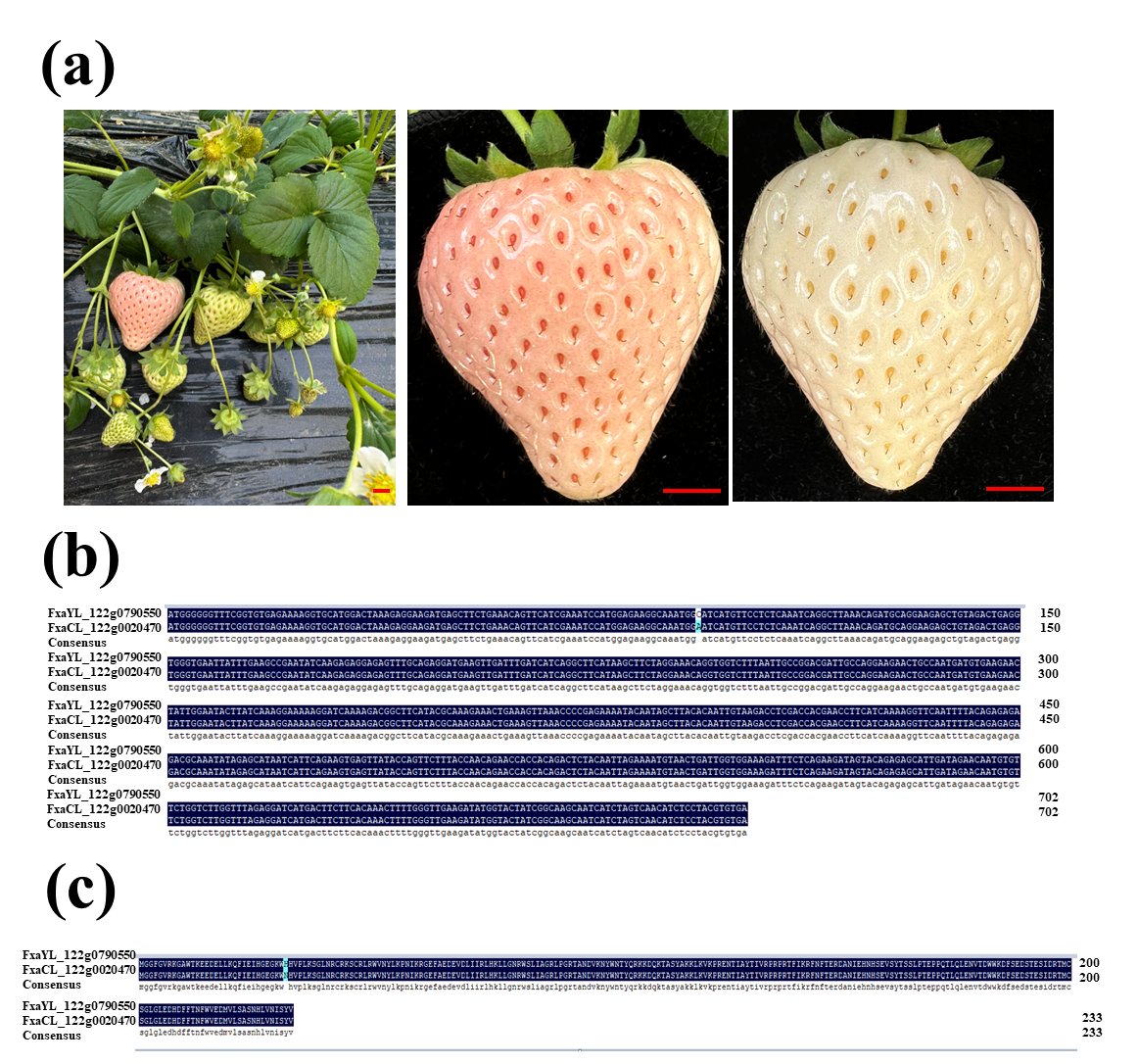


**Figure S1. The phenotypes of ‘Chulian’ strawberry and the comparisons of the coding region and amino acids of FaMYB10 on chr1-2-2 from ‘Chulian’ and ‘Yanli’ strawberry.**

(a) The phenotypes of ‘Chulian’ strawberry. Left: ‘Chulian’ strawberry in the greenhouse; Middle: The front of ‘Chulian’ strawberry; Right: The back of the ‘Chulian’ strawberry. (b) The comparison of the coding region of *FaMYB10* on chr1-2-2 from ‘Chulian’ and ‘Yanli’ strawberry; (c) The comparison of the amino acids of FaMYB10 on chr1-2-2 from ‘Chulian’ and ‘Yanli’ strawberry. FxaYL_122g0790550 is FaMYB10 on chr1-2-2 of ‘Yanli’ strawberry; FxaCL_122g0020470 is FaMYB10 on chr1-2-2 of ‘Chulian strawberry.


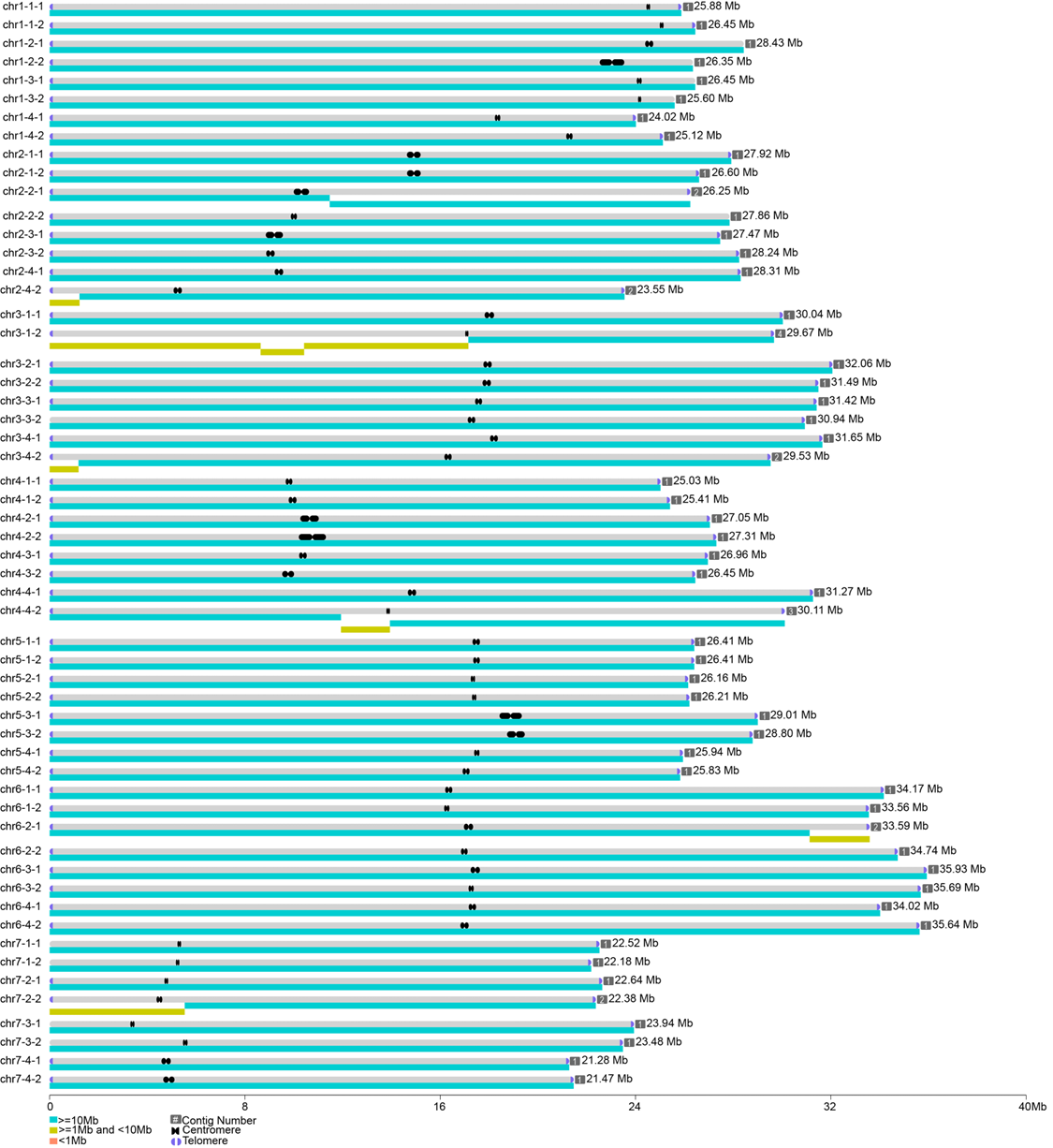


**Figure S2. The contig numbers, telomeres, and centromeres of ‘Chulian’ strawberry genome.**

**
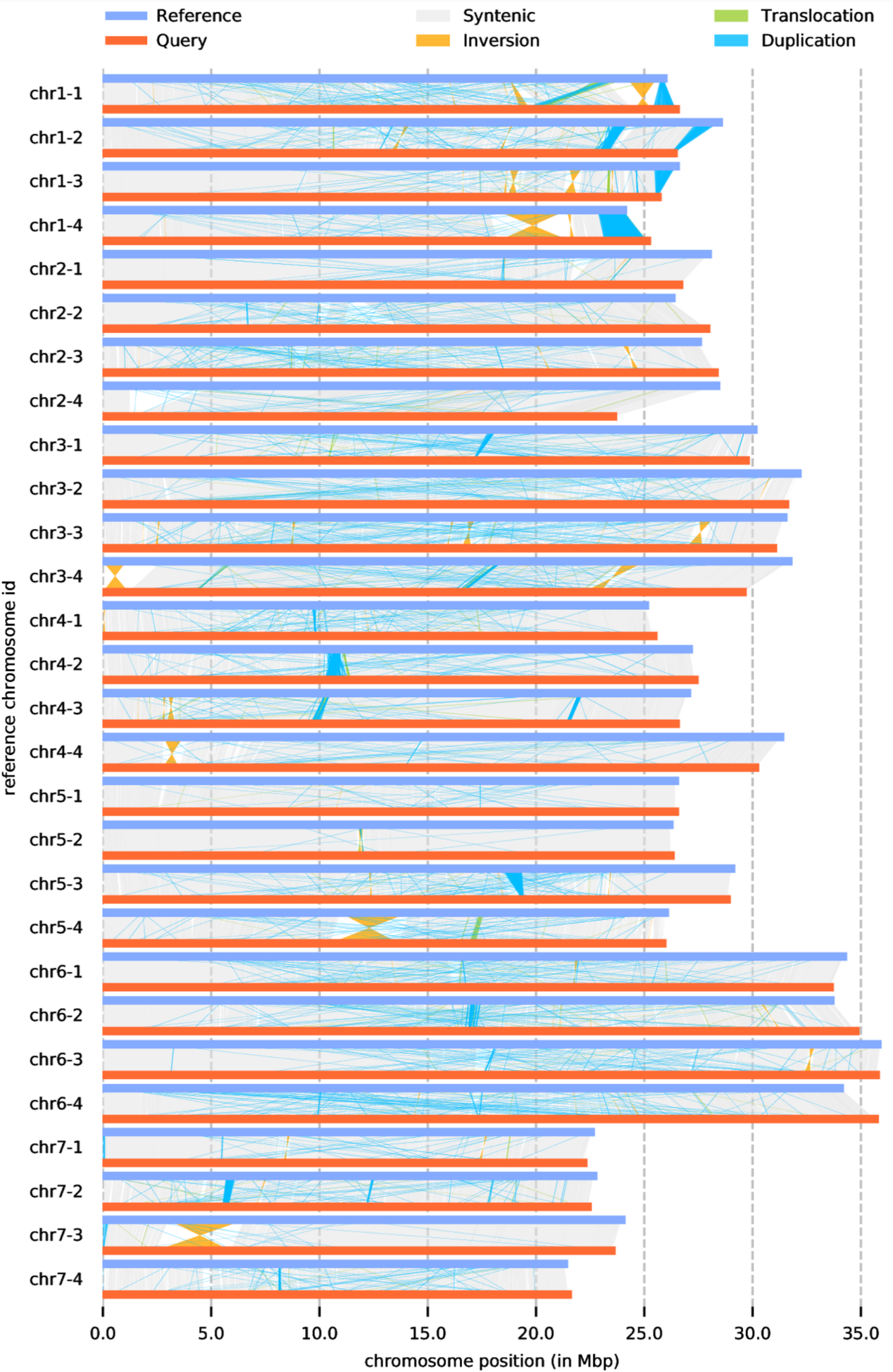
 Figure S3.** **The collinearity of Hap1 and Hap2 of ‘Chulian’ strawberry genome.**

Reference: Hap1 genome of ‘Chulian’ strawberry; Query: Hap2 genome of ‘Chulian’ strawberry.

**
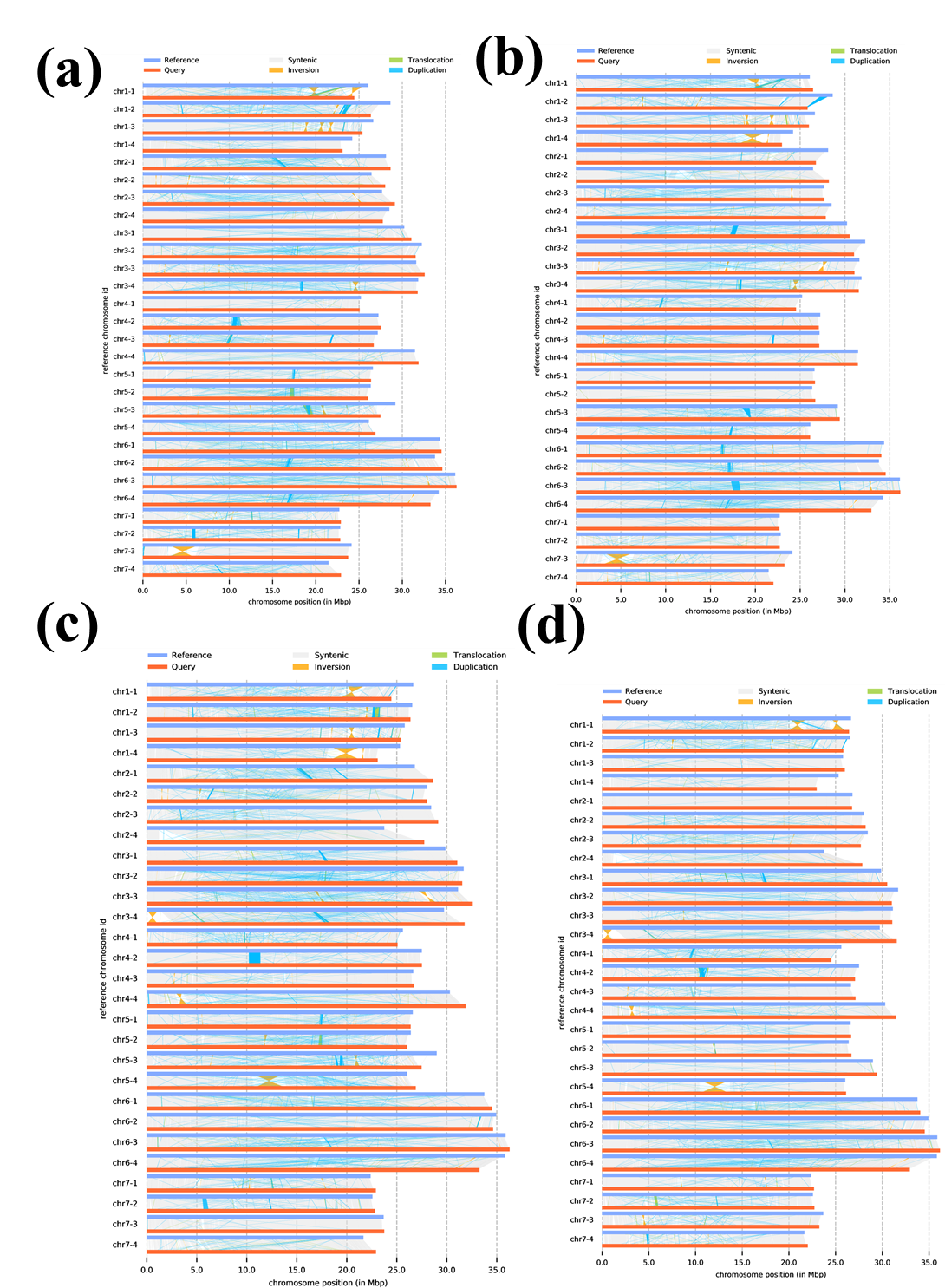
**

**Figure S4. The collinearity of ‘Chulian’ and ‘Yanli’ strawberry genome.**

1. The collinearity of Hap1 of ‘Chulian’ and Hap1 of ‘Yanli’ strawberry genome; (b) The collinearity of Hap1 of ‘Chulian’ and Hap2 of ‘Yanli’ strawberry genome; (c) The collinearity of Hap2 of ‘Chulian’ and Hap1 of ‘Yanli’ strawberry genome; (d) The collinearity of Hap2 of ‘Chulian’ and Hap2 of ‘Yanli’ strawberry genome. Reference: ‘Chulian’ strawberry; Query: ‘Yanli’ strawberry.


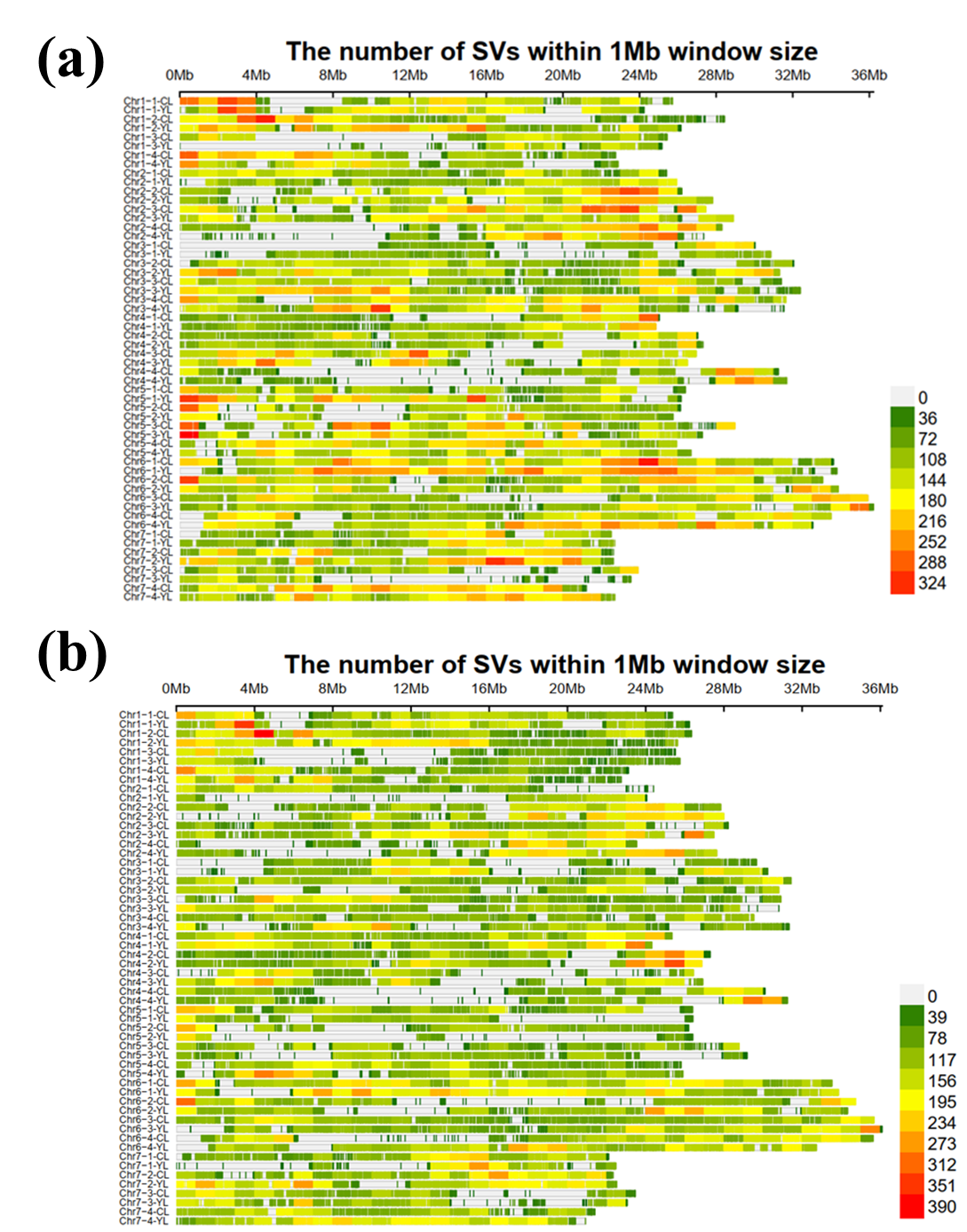


**Figure S5. The structural variations (SVs) between the ‘Chulian’ and ‘Yanli’ strawberry genome.**

(a) The SVs in the Hap1 of ‘Chulian’ compared with ‘Yanli’ strawberry; (b) The SVs in the Hap2 of ‘Chulian’ compared with ‘Yanli’ strawberry. ‘CL’ represents ‘Chulian’ strawberry; ‘YL’ represents ‘Yanli strawberry.


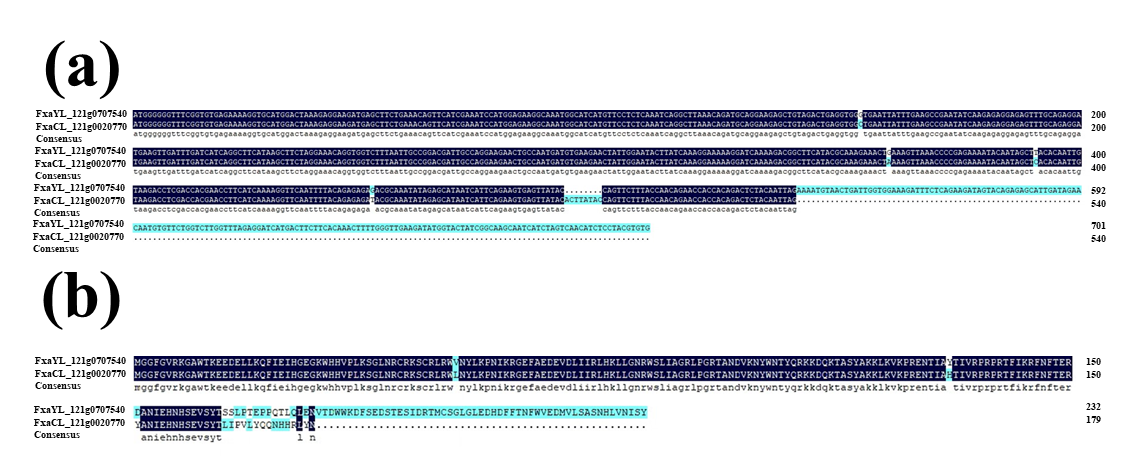


**Figure S6. The comparisons of the coding region and amino acids of FaMYB10 on chr1-2-1 from ‘Chulian’ and ‘Yanli’ strawberry.**

(a) The comparison of the coding region of *FaMYB10* on chr1-2-1 from ‘Chulian’ and ‘Yanli’ strawberry; (b) The comparison of the amino acids of FaMYB10 on chr1-2-1 from ‘Chulian’ and ‘Yanli’ strawberry. FxaYL_121g0707540 is FaMYB10 on chr1-2-1 of ‘Yanli’ strawberry; FxaCL_121g0020770 is FaMYB10 on chr1-2-1 of ‘Chulian strawberry.


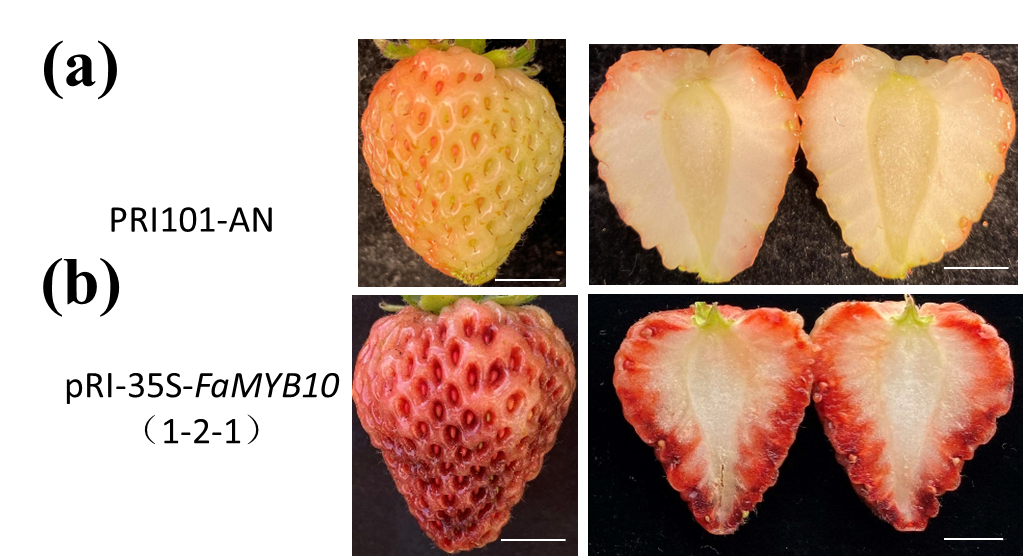


**Figure S7. The transient functional analysis of overexpression of *FaMYB10* on chr1-2-1 of ‘Yanli’ with CaMV 35S promoter on the fruits of ‘Chulian’.**


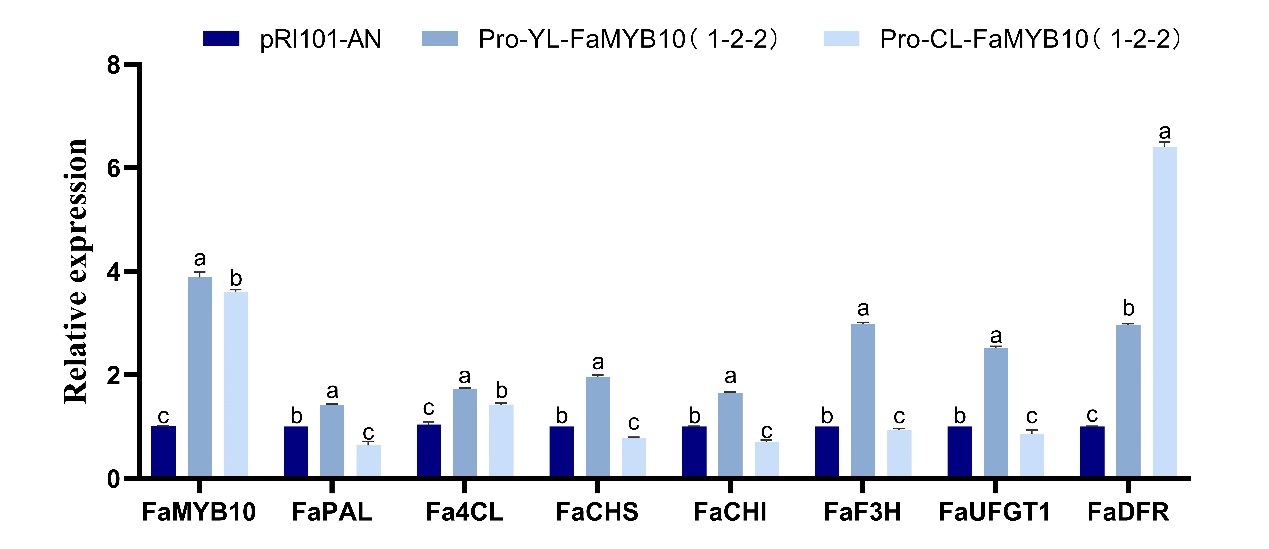


**Figure S8. The expression of anthocyanin biosynthetic genes between the fruits of** **importing *FaMYB10* on chr1-2-2 of ‘Yanli’ with its promoter [Pro-YL-FaMYB10(1-2-2)] and importing *FaMYB10* on chr1-2-2 of ‘Chulian’ with its promoter [Pro-CL-FaMYB10(1-2-2)].**

**
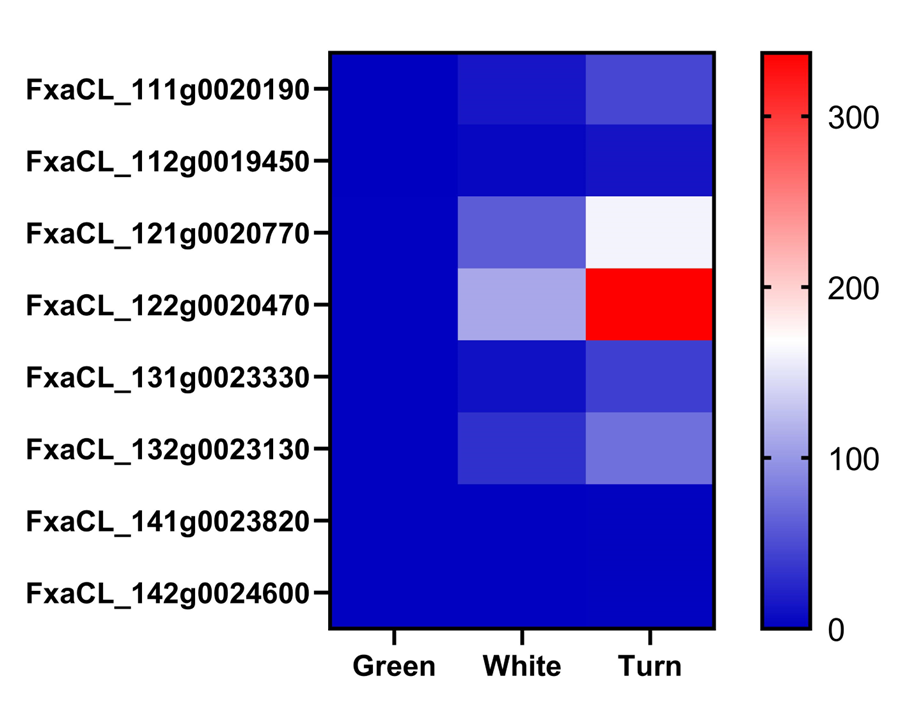
**

**Figure S9. The expression of *FaMYB10* from ‘Chulian’ strawberry fruit at green, white, and turn stage.**

FxaCL_111g0020190 is *FaMYB10* on chr1-1-1 of ‘Chulian’ strawberry; FxaCL_112g00119450 is *FaMYB10* on chr1-1-2 of ‘Chulian’ strawberry; FxaCL_121g0020770 is *FaMYB10* on chr1-2-1 of ‘Chulian’ strawberry. FxaCL_122g0020470 is *FaMYB10* on chr1-2-2 of ‘Chulian’ strawberry; FxaCL_131g0023330 is *FaMYB10* on chr1-3-1 of ‘Chulian’ strawberry; FxaCL_132g0023130 is *FaMYB10* on chr1-3-2 of ‘Chulian’ strawberry; FxaCL_141g0023820 is *FaMYB10* on chr1-4-1 of ‘Chulian’ strawberry; FxaCL_142g0024600 is *FaMYB10* on chr1-4-2 of ‘Chulian’ strawberry.

**
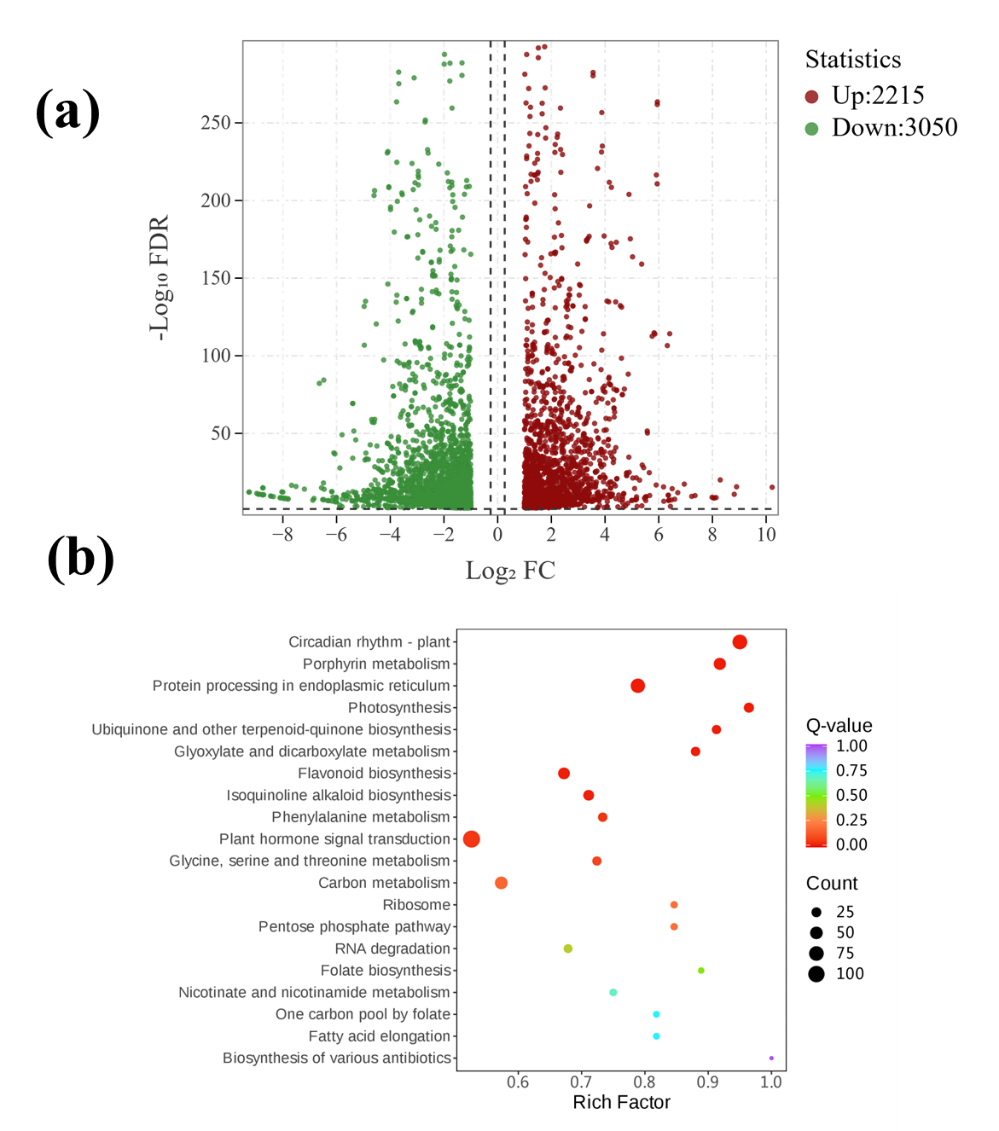
**

**Figure S10. The volcano map and KEGG of differentially expressed genes of ‘Chulian’ strawberry fruit under lighting and shading.**

(a) The volcano map of differentially expressed genes of ‘Chulian’ strawberry fruit under lighting and shading; (b) The KEGG of differentially expressed genes of ‘Chulian’ strawberry fruit under lighting and shading.


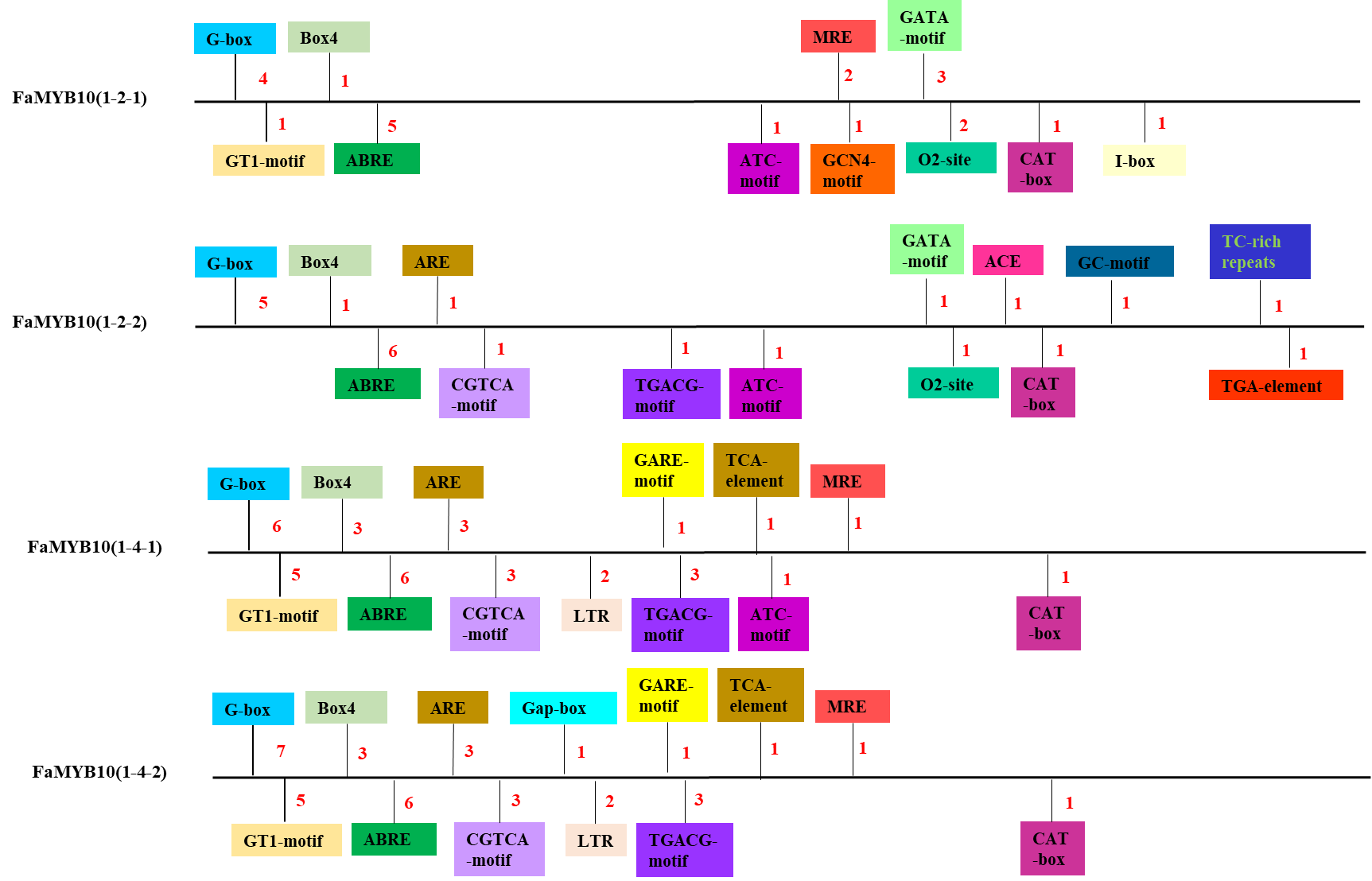


**Figure S11. The schematic figure of cis-elements differences in the promoter region between *FaMYB10* on chr1-2 and *FaMYB10* on chr1-4 of ‘Chulian’ strawberry under lighting and shading treatment.**

**Table S1. The** **Centromere information of the ‘Chulian’ strawberry genome.**

|  | Hap1 |  | Hap2 |  |
| --- | --- | --- | --- | --- |
| Chromosome | Start | End | Start | End |
| chr1-1 | 24,449,164 | 24,597,016 | 24,993,950 | 25,145,862 |
| chr1-2 | 24,397,643 | 24,726,160 | 22,535,148 | 23,540,660 |
| chr1-3 | 24,051,332 | 24,251,471 | 24,105,768 | 24,231,234 |
| chr1-4 | 18,242,208 | 18,457,483 | 21,164,620 | 21,421,828 |
| chr2-1 | 14,637,723 | 15,194,287 | 14,642,920 | 15,199,191 |
| chr2-2 | 9,999,548 | 10,624,089 | 9,883,739 | 10,126,354 |
| chr2-3 | 8,855,676 | 9,554,495 | 8,872,397 | 9,215,510 |
| chr2-4 | 9,220,058 | 9,565,727 | 5,076,809 | 5,414,990 |
| chr3-1 | 17,820,923 | 18,204,305 | 17,028,530 | 17,154,850 |
| chr3-2 | 17,769,514 | 18,107,283 | 17,736,240 | 18,074,009 |
| chr3-3 | 17,431,165 | 17,713,309 | 17,129,958 | 17,434,764 |
| chr3-4 | 18,042,780 | 18,359,445 | 16,173,813 | 16,467,116 |
| chr4-1 | 9,668,596 | 9,941,698 | 9,791,865 | 10,113,179 |
| chr4-2 | 10,269,397 | 11,024,644 | 10,207,237 | 11,316,853 |
| chr4-3 | 10,221,807 | 10,530,469 | 9,528,135 | 10,010,625 |
| chr4-4 | 14,676,674 | 15,012,209 | 13,795,548 | 13,937,639 |
| chr5-1 | 17,324,880 | 17,626,207 | 17,354,777 | 17,620,042 |
| chr5-2 | 17,253,520 | 17,427,080 | 17,304,390 | 17,477,942 |
| chr5-3 | 18,429,445 | 19,339,549 | 18,740,155 | 19,461,781 |
| chr5-4 | 17,391,295 | 17,607,359 | 16,917,786 | 17,202,579 |
| chr6-1 | 16,205,145 | 16,493,757 | 16,162,163 | 16,374,730 |
| chr6-2 | 16,971,109 | 17,344,916 | 16,852,062 | 17,118,877 |
| chr6-3 | 17,247,883 | 17,625,710 | 17,162,520 | 17,369,954 |
| chr6-4 | 17,167,991 | 17,468,255 | 16,828,161 | 17,163,040 |
| chr7-1 | 5,237,384 | 5,382,765 | 5,172,232 | 5,314,576 |
| chr7-2 | 4,703,839 | 4,855,049 | 4,380,178 | 4,612,095 |
| chr7-3 | 3,306,646 | 3,474,995 | 5,460,684 | 5,648,371 |
| chr7-4 | 4,582,642 | 4,960,804 | 4,655,406 | 5,114,556 |

**Table S2. The telomere information of the ‘Chulian’ strawberry genome.**

| Chromosome | Hap1 Start repeat unit number | Hap1 End repeat unit number | Hap2 Start repeat unit number | Hap2 End repeat unit number | |
| --- | --- | --- | --- | --- | --- |
| chr1-1 | 188 | 166 | 188 | 138 |  |
| chr1-2 | 165 | 0 | 159 | 0 |  |
| chr1-3 | 188 | 0 | 188 | 0 |  |
| chr1-4 | 144 | 6 | 174 | 168 |  |
| chr2-1 | 167 | 192 | 167 | 192 |  |
| chr2-2 | 226 | 207 | 226 | 0 |  |
| chr2-3 | 182 | 150 | 184 | 142 |  |
| chr2-4 | 161 | 378 | 189 | 202 |  |
| chr3-1 | 197 | 167 | 197 | 195 |  |
| chr3-2 | 206 | 172 | 182 | 190 |  |
| chr3-3 | 176 | 160 | 0 | 190 |  |
| chr3-4 | 166 | 190 | 188 | 166 |  |
| chr4-1 | 176 | 186 | 130 | 170 |  |
| chr4-2 | 165 | 194 | 181 | 180 |  |
| chr4-3 | 167 | 200 | 213 | 154 |  |
| chr4-4 | 178 | 321 | 159 | 200 |  |
| chr5-1 | 176 | 170 | 160 | 168 |  |
| chr5-2 | 191 | 202 | 164 | 202 |  |
| chr5-3 | 193 | 194 | 189 | 125 |  |
| chr5-4 | 164 | 184 | 144 | 188 |  |
| chr6-1 | 183 | 173 | 150 | 173 |  |
| chr6-2 | 206 | 109 | 206 | 175 |  |
| chr6-3 | 143 | 187 | 191 | 187 |  |
| chr6-4 | 157 | 152 | 157 | 183 |  |
| chr7-1 | 0 | 163 | 0 | 176 |  |
| chr7-2 | 193 | 170 | 186 | 180 |  |
| chr7-3 | 0 | 170 | 0 | 183 |  |
| chr7-4 | 209 | 52 | 190 | 52 |  |

**Table S3. Genome assembly integrity assessment of Hap1 of ‘Chulian’ strawberry by** **BUSCO.**

| Type | BUSCOs num | Percentage (%) |
| --- | --- | --- |
| Complete BUSCOs (C) | 1,598 | 99.01 |
| Complete and single-copy BUSCOs (S) | 49 | 3.04 |
| Complete and duplicated BUSCOs (D) | 1,549 | 95.97 |
| Fragmented BUSCOs (F) | 2 | 0.12 |
| Missing BUSCOs (M) | 14 | 0.87 |
| Total BUSCO groups searched | 1,614 | 100 |

**Table S4. Genome assembly integrity assessment of Hap2 of ‘Chulian’ strawberry by** **BUSCO.**

| Type | BUSCOs num | Percentage (%) |
| --- | --- | --- |
| Complete BUSCOs (C) | 1,602 | 99.26 |
| Complete and single-copy BUSCOs (S) | 53 | 3.28 |
| Complete and duplicated BUSCOs (D) | 1,549 | 95.97 |
| Fragmented BUSCOs (F) | 1 | 0.06 |
| Missing BUSCOs (M) | 11 | 0.68 |
| Total BUSCO groups searched | 1,614 | 100 |

**Table S5. The information on repetitive sequences of Hap1 of ‘Chulian’ strawberry**

|  | TEs based on RepeatMasker |  | TE based on RepeatProteinMask |  | De novo |  | Combined TEs |  |
| --- | --- | --- | --- | --- | --- | --- | --- | --- |
| TE types | Length (bp) | % in Genome | Length (bp) | % in Genome | Length (bp) | % in Genome | Length (bp) | % in Genome |
| DNA | 75,297,948 | 9.56 | 16,051,717 | 2.04 | 64,543,941 | 8.19 | 96,715,659 | 12.28 |
| LINE | 8,231,680 | 1.04 | 3,913,525 | 0.5 | 9,803,999 | 1.24 | 13,585,564 | 1.72 |
| SINE | 26,341 | 0 | 0 | 0 | 101,727 | 0.01 | 128,068 | 0.02 |
| LTR | 119,833,545 | 15.21 | 48,404,896 | 6.14 | 119,951,164 | 15.23 | 175,608,959 | 22.29 |
| Satellite | 889,164 | 0.11 | 0 | 0 | 923,169 | 0.12 | 1,441,383 | 0.18 |
| Simple_repeat | 0 | 0 | 0 | 0 | 29,455 | 0 | 29,455 | 0 |
| Other | 805 | 0 | 792 | 0 | 0 | 0 | 1,597 | 0 |
| Unknown | 984,724 | 0.12 | 32,787 | 0 | 30,388,737 | 3.86 | 30,756,748 | 3.9 |
| Total | 203,846,614 | 25.87 | 68,387,249 | 8.68 | 223,974,315 | 28.43 | 312,946,577 | 39.72 |

**Table S6. The information on repetitive sequences of Hap2 of ‘Chulian’ strawberry**

|  | TEs based on RepeatMasker |  | TE based on RepeatProteinMask |  | De novo |  | Combined TEs |  |
| --- | --- | --- | --- | --- | --- | --- | --- | --- |
| TE types | Length (bp) | % in Genome | Length (bp) | % in Genome | Length (bp) | % in Genome | Length (bp) | % in Genome |
| DNA | 74,022,781 | 9.51 | 15,700,237 | 2.02 | 62,379,872 | 8.02 | 96,169,839 | 12.36 |
| LINE | 8,058,196 | 1.04 | 3,754,407 | 0.48 | 8,739,381 | 1.12 | 12,866,696 | 1.65 |
| SINE | 25,534 | 0 | 0 | 0 | 32,993 | 0 | 58,527 | 0.01 |
| LTR | 117,985,095 | 15.16 | 47,507,023 | 6.11 | 119,205,358 | 15.32 | 175,953,274 | 22.61 |
| Satellite | 756,569 | 0.1 | 0 | 0 | 1,020,273 | 0.13 | 1,424,865 | 0.18 |
| Simple_repeat | 0 | 0 | 0 | 0 | 60,332 | 0.01 | 60,332 | 0.01 |
| Other | 758 | 0 | 792 | 0 | 0 | 0 | 1,550 | 0 |
| Unknown | 989,031 | 0.13 | 31,677 | 0 | 29,445,674 | 3.78 | 29,892,196 | 3.84 |
| Total | 200,558,599 | 25.78 | 66,978,540 | 8.61 | 218,097,095 | 28.03 | 309,671,494 | 39.8 |

**Table S7. The structural variations between Hap1 and Hap2 of ‘Chulian’ strawberry Genome.**

| Variation type | Number | Length of Reference | Length of Query |
| --- | --- | --- | --- |
| Syntenic regions | 16,315 | 630,943,319 | 630,835,211 |
| Inversions | 155 | 21,126,401 | 20,997,919 |
| Translocations | 3,830 | 49,838,854 | 50,001,751 |
| Duplications (reference) | 12,540 | 104,603,808 | - |
| Duplications (query) | 9,116 | - | 64,645,198 |
| Not aligned (reference) | 16,044 | 69,888,918 | - |
| Not aligned (query) | 15,824 | - | 65,792,008 |

Reference: Hap1 of ‘Chulian’ strawberry Genome; Query: Hap2 of ‘Chulian’ strawberry Genome.

**Table S8. The cis-elements differences in the promoter region between *FaMYB10* on chr1-2 and *FaMYB10* on chr1-4 of ‘Chulian’ strawberry under lighting and shading treatment.**

| Cis-elements name | Cis-elements | FaMYB10 | FaMYB10 | FaMYB10 | FaMYB10 | Cis-elements function |
| --- | --- | --- | --- | --- | --- | --- |
|  | sequence | 1-2-1 | 1-2-2 | 1-4-1 | 1-4-2 |  |
| G-box | CACGTC, | 4 | 5 | 6 | 7 | cis-acting regulatory element involved in light responsiveness |
|  | CACGTT, |  |  |  |  |  |
|  | TAAACGTG |  |  |  |  |  |
| GT1-motif | GGTTAA | 1 | 0 | 5 | 5 | light responsive element |
| Box 4 | ATTAAT | 1 | 1 | 3 | 3 | part of a conserved DNA module involved in light responsiveness |
| ABRE | ACGTG | 5 | 6 | 6 | 6 | cis-acting element involved in the abscisic acid responsiveness |
| ARE | AAACCA | 0 | 1 | 3 | 3 | cis-acting regulatory element essential for the anaerobic induction |
| CGTCA-motif | CGTCA | 0 | 1 | 3 | 3 | cis-acting regulatory element involved in the MeJA-responsiveness |
| Gap-box | CAAATGAA(A/G)A | 0 | 0 | 0 | 1 | part of a light responsive element |
| GARE-motif | TCTGTTG | 0 | 0 | 1 | 1 | gibberellin-responsive element |
| LTR | CCGAAA | 0 | 0 | 2 | 2 | cis-acting element involved in low-temperature responsiveness |
| TCA-element | CCATCTTTTT | 0 | 0 | 1 | 1 | cis-acting element involved in salicylic acid responsiveness |
| TGACG-motif | TGACG | 0 | 1 | 3 | 3 | cis-acting regulatory element involved in the MeJA-responsiveness |
| MRE | AACCTAA | 2 | 0 | 1 | 1 | MYB binding site involved in light responsiveness |
| ATC-motif | TGCTATCCA | 1 | 1 | 1 | 0 | part of a conserved DNA module involved in light responsiveness |
| GCN4_motif | TGAGTCA | 1 | 0 | 0 | 0 | cis-regulatory element involved in endosperm expression |
| GATA-motif | GATAGGA | 3 | 1 | 0 | 0 | part of a light responsive element |
| O2-site | GATGACATGG | 2 | 1 | 0 | 0 | cis-acting regulatory element involved in zein metabolism regulation |
| ACE | CTAACGTATT | 0 | 1 | 0 | 0 | cis-acting element involved in light responsiveness |
| CAT-box | GCCACT | 1 | 1 | 1 | 1 | cis-acting regulatory element related to meristem expression |
| GC-motif | CCCCCG | 0 | 1 | 0 | 0 | enhancer-like element involved in anoxic specific inducibility |
| I-box | gGATAAGGTG | 1 | 0 | 0 | 0 | part of a light responsive element |
| TC-rich repeats | GTTTTCTTAC | 0 | 1 | 0 | 0 | cis-acting element involved in defense and stress responsiveness |
| TGA-element | AACGAC | 0 | 1 | 0 | 0 | auxin-responsive element |
|  | Total | 22 | 23 | 36 | 37 |  |

The gene accession of FaMYB10(1-2-1) is FxaCL_121g0020770; The gene accession of FaMYB10 is FxaCL_122g0020470; The gene accession of FaMYB10(1-4-1) is FxaCL_141g0023820; The gene accession of FaMYB10(1-4-2) is FxaCL_142g0024600.
